# Habituation to Feedback Delay Restores Degraded Visuomotor Adaptation by Altering Both Sensory Prediction Error and the Sensitivity of Adaptation to the Error

**DOI:** 10.3389/fpsyg.2012.00540

**Published:** 2012-12-14

**Authors:** Takuya Honda, Masaya Hirashima, Daichi Nozaki

**Affiliations:** ^1^Graduate School of Education, The University of TokyoTokyo, Japan; ^2^Japan Society for the Promotion of ScienceTokyo, Japan; ^3^Advanced Telecommunications Research Institute InternationalSeika-cho, Japan

**Keywords:** sensorimotor association, feedback delay, delay adaptation, visuomotor adaptation, forward model

## Abstract

Sensory prediction error, which is the difference between actual and predicted sensory consequences, is a driving force of motor learning. Thus, appropriate temporal associations between the actual sensory feedback signals and motor commands for predicting sensory consequences are crucial for the brain to calculate the sensory prediction error accurately. Indeed, it has been shown that artificially introduced delays in visual feedback degrade motor learning. However, our previous study has showed that degraded adaptation is alleviated by prior habituation to the delay. Here, we investigate how the motor learning system accomplishes this alleviation. After the subjects habituated reaching movements in either 0- or 200-ms delayed cursor, visual rotation of 10° was imposed to the cursor with varying delay (0, 100, 200, or 300 ms) with each delay imposed in at least 1 out of 5–6 trials. Then, the aftereffect in the next trial was quantified to evaluate the adaptation response. After habituation to the 0-ms delayed cursor, the adaptation response was maximal when the visual feedback of the perturbation was provided with 0-ms delay and gradually decreased as the delay increased. On the other hand, habituation to the 200-ms delayed cursor alleviated the degraded adaptation response to the visual perturbation imposed during the 200-ms and longer delay (300 ms). However, habituation did not affect the adaptation response to the visual perturbation imposed during delays (0- and 100-ms delay) shorter than the habituated delay (200 ms). These results may be explained by assuming that habituation to the delayed feedback not only shifts the position of the hand predicted by motor command toward the delayed cursor positions, but also increases the degree to which the brain uses a certain amount of sensory prediction error to correct a motor command.

## Introduction

How the brain associates an action with its sensory consequence is crucial to sensorimotor learning. It is widely accepted that the brain predicts the sensory consequence of a motor command using a predictive model of the motor apparatus (i.e., internal forward model) before the actual sensory feedback signals become available (Wolpert et al., 1995; Shadmehr and Krakauer, 2008). The forward model is considered to play an important role in achieving fast and accurate control of movement without depending on delayed sensory feedback (Guthrie et al., 1983; Cooke and Diggles, 1984; Bard et al., 1999; Xu-Wilson et al., 2009). To maintain accurate prediction by the forward model, the brain must update the forward model according to the sensory prediction error between the predicted and actual sensory consequences (Held and Freedman, 1963; Mazzoni and Krakauer, 2006; Tseng et al., 2007).

In this motor adaptation scheme, the temporal relationship between actions and their sensory consequences is important to evaluate the sensory prediction error accurately (Ikegami et al., 2012). Kitazawa et al. (1995), by using prism adaptation during reaching movements, showed that motor adaptation is degraded when the location of the reaching endpoint is displayed with an artificial delay. However, we have the ability to perform motor actions even in the presence of a feedback delay that may change due to several factors (e.g., body growth or manipulating tool). Furthermore, recent psychophysical studies have demonstrated that when subjects experienced a constant delay between an action and its sensory consequence, the delayed sensory consequences came to be perceived as shifted backward in time toward their actions (Haggard et al., 2002; Haggard and Clark, 2003; Park et al., 2003; Stetson et al., 2006; Heron et al., 2009). Considering these points, it is reasonable to assume that the sensorimotor system can also adapt to a wide variety of delays.

Indeed, we recently showed that, when visual feedback of the hand position is provided by a cursor throughout an entire reaching trajectory, the decreased rate of motor adaptation to a visual rotation caused by delayed feedback is alleviated by prior repeated exposure to the delayed cursor (Honda et al., 2012). This result suggests that the ability of visuomotor adaptation can be influenced by habituation to the delayed feedback.

Computational studies have suggested that when a certain amount of sensory prediction error is experienced, the motor command for the subsequent trial is corrected in proportion to the amount of this sensory prediction error (Shadmehr et al., 2010). Thus, one possible factor for the alleviation is that repeated exposure to the delayed cursor increases the proportional coefficient, or the sensitivity of adaptation to the sensory prediction error, as we demonstrated in our previous study (Honda et al., 2012).

**Figure 1:**
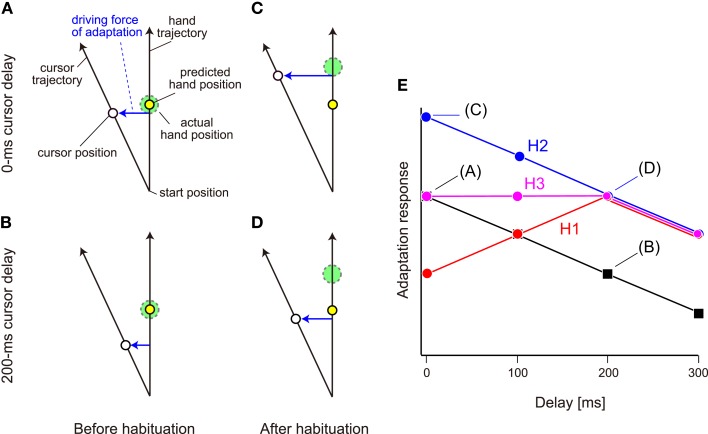
**Schematic representation of the hypotheses**. **(A)** We assume that the sensory prediction error represented by lateral deviation from the hand position predicted by the forward model to the cursor is a driving force of adaptation to a visual rotation. **(B)** The sensory prediction error decreases when a cursor delay of 200 ms is introduced. In **(A, B)**, the predicted hand position (broken circle) is identical to the actual hand position (continuous circle). **(C,D)** However, if the habituation to delayed cursor of 200 ms shifts the predicted hand position to the delayed position, the amount of sensory prediction error is restored when the cursor is displayed with the same 200 ms delay **(D)** and it rather increases when the cursor is displayed with no-delay **(C)**. **(E)** When no visual feedback delay is introduced, the adaptation response to the visual rotation provided with a certain delay should gradually decrease with the increase in delay. Three hypotheses predict different patterns of adaptation response after habituation to a visual feedback delay of 200 ms. Hypothesis H1 predicts the response is maximal at 200 ms delay (red), while the H2 predicts the response has a monotonically decreasing function (blue), and H3 predicts an intermediate response of H1 and H2 (magenta).

However, habituation to the delayed cursor condition can alter not only the sensitivity but also the degree of the sensory prediction error itself. Figures 1A–D illustrate the positions of the actual hand and cursor when the predicted hand position reaches the middle position. Before habituation to the delayed cursor condition, the predicted hand is located at almost the same position as the actual hand (Figures 1A,B). Here, in order to explain the possible changes in the sensory prediction error with the cursor delay, we assume that when visual rotation is imposed, the sensory prediction error is a lateral deviation from the predicted hand position to the cursor. If the cursor is suddenly delayed (i.e., before habituation), the sensory prediction error caused by the visual rotation should be smaller than that of the *no-delay condition* (Figure 1B vs. Figure 1A). On the other hand, after habituation to the delay, the predicted hand position is shifted toward the past hand position (Figures 1C,D). This shift could recover the amount of sensory prediction error for the delayed cursor condition (Figure 1D vs. Figure 1B). At the same time, however, the sensory prediction error could be larger than reality if the delay is removed suddenly (Figure 1C vs. Figure 1D).

Thus, we have three alternative hypotheses, namely, increase in the sensitivity (hypothesis H1), shift of the predicted hand position (hypothesis H2), or both (hypothesis H3). To test these hypotheses, we systematically examined the adaptation response of reaching movements to visual rotation applied with varying delays after repeated exposure to 0-ms (*no-delay condition*) or 200-ms delayed cursor (*delay condition*). In the *no-delay condition*, as shown in previous studies (Kitazawa et al., 1995; Honda et al., 2012), all three hypotheses predicted the same outcome: the adaptation response should gradually decrease with the delay time (Figure 1E; black). In the *delay condition*, however, the three hypotheses predicted different patterns. Hypothesis H1 predicted that the adaptation response is the maximum when visual perturbation is applied at a 200-ms delay, and the response to the 0-, 100-, and 300-ms delay becomes minor, because the sensitivity of visuomotor adaptation is optimized to this delay (Figure 1E; red). Hypothesis H2 also predicted that after habituation to the delayed cursor, the adaptation response to the visual perturbation provided with a 200-ms delay is recovered (Figure 1E; blue) because the sensory prediction error is recovered (Figure 1D vs. Figure 1B). However, after the shift of the predicted hand position by habituation to the delay, the sensory prediction error is still greatest for the 0-ms delay (Figure 1C), and gradually decreases with increasing delay (Figures 1C,D). Therefore, we should observe a monotonic decrease in the adaptation response with increasing delay (Figure 1E; blue). Hypothesis H3 predicts an intermediate adaptation response (Figure 1E; magenta). We investigated which alternative hypothesis was likely to explain the data.

## Materials and Methods

### Participants

Twenty-seven volunteers (15 men and 12 women; age range, 19–30 years) participated in this study. Participants had no cognitive or motor disorders, and were naïve to the visuomotor rotation task and purpose of the experiment. Their dominant hands were determined by the Edinburgh Handedness Inventory (Oldfield, 1971); all participants were right-handed. Further, they were paid for their time. This study was conducted in accordance with the Declaration of Helsinki and the experimental procedures were approved by the ethics committee of the Graduate School of Education at the University of Tokyo. Written informed consent was obtained from all participants prior to performing the experiments.

### Apparatus and motor task

**Figure 2:**
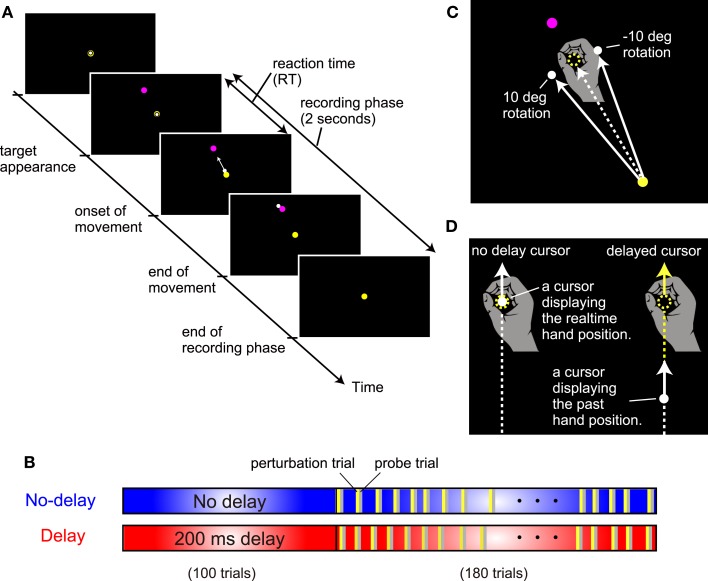
**Experimental setting. (A)** The sequence of events in a trial. **(B)** Two experimental conditions for the cursor display. In the *no-delay condition*, there was no artificial delay (blue box) between the hand and cursor in the simple reaching task. In the *delay condition*, there was an artificial delay of 200 ms between the hand and cursor display of simple reaching. **(C)** Visuomotor rotation. The direction of the cursor was rotated from the direction of the hand around the starting position clockwise or counterclockwise at 10°. **(D)** Types of cursors. An artificial delay between the hand and cursor display was chosen from 0, 100, 200, or 300 ms.

Participants sat on a straight-backed chair while grasping the handle of a robotic manipulandum with their right hand (Phantom Premium 1.5HF, SensAble Technologies, USA). A spring simulated by the device (1.0 N/mm) generated a virtual horizontal plane on which the handle movement was restricted. A projector was used to display the position of the handle with a white circle cursor (diameter, 8 mm) on a horizontal screen (size, 45 cm × 60 cm) placed at 13 cm above the virtual plane and 10–15 cm below the shoulder level. Thus, the screen board prevented the participants from directly seeing their arm and the handle. Before each trial, participants were required to hold the cursor in its starting position (10-mm diameter, yellow circle; Figure 2A). After a 2-s holding time, a target (10-mm diameter, magenta circle) appeared at 30° counterclockwise from straight ahead (only one target was used in this study), signaling the participant to initiate a reaching movement. The starting position was located approximately 25 cm in front of the body. The distance between the starting position and the target was 10 cm. Participants were required to move the handle with a peak velocity (PV) in the range between 350 to 550 mm/s. A warning message appeared on the screen if the movement velocity of the handle rose above (“Fast”) or fell below (“Slow”) this threshold value. After the completion of each trial (i.e., after the cursor stopped), the handle was automatically moved back to the starting position by the manipulandum; during this time, the cursor disappeared and remained invisible until the handle reached the starting position. Visual feedback of the cursor during the reaching movement was always provided, except in the probe trials. The starting position was always visible. The target was extinguished after the reaching movement was completed. The position and velocity of the handle were recorded with a sampling frequency of 500 Hz for offline analysis.

### Instructions

Participants were instructed to move the cursor from the start position to the target with a straight and uncorrected stroke (Mazzoni and Krakauer, 2006; Ikegami et al., 2010), and to initiate the reaching movement as soon as the target appeared. After each stroke, they were instructed to maintain the hand position where it stopped and not to correct the position even if the cursor was not on the target. Such uncorrected strokes were adopted to eliminate the possible effect of online correction of the current trial’s movement to the adaptation response in the next trial (The absence of online correction was confirmed: we did not observe a significant amount of correction in the angular position of the hand relative to the starting position at its PV and at the movement offset). No performance-based rewards such as money or sounds were provided after each trial.

### Experimental conditions

To examine the mechanism by which the motor learning system uses for the adaptation to an artificially introduced delay, the present study systematically investigated the adaptation response to a visual perturbation applied with a varying delay in the *no-delay condition* and *delay condition* (Figure 2B).

In the *no-delay condition*, participants performed normal reaching movements in which no-delay was introduced between the actual hand and cursor movements. After the initial 100 trials for habituation to the no-delay cursor, visual perturbation trials were randomly interleaved once for every 5–6 trials during the subsequent 180 trials (see yellow bars in Figure 2B). In the perturbation trials (24 trials), the cursor’s movement direction from the start position was rotated by 10° clockwise (12 trials) or counterclockwise (12 trials) from the hand’s movement direction (Figure 2C). In the perturbation trials, visual feedback delay was also manipulated with a delay of 0-, 100-, 200-, or 300-ms that was artificially introduced between the cursor and the hand position, i.e., the position of the cursor displayed the hand position that had occurred 0, 100, 200, or 300 ms before, respectively (Figure 2D). Thus, the 24 perturbation trials consisted of three trials for each of the eight cursor manipulations (2 rotation directions × 4 cursor delays). If this visual perturbation induced adaptation, then participants would move the handle in the opposite direction (i.e., aftereffect) in the next trial (probe trial; see gray bars in Figure 2B). We quantified the aftereffects by measuring the movement direction during the probe trials (see Data Analysis). Further, during the probe trials, no visual feedback of the cursor was provided to remove the effect of online visual feedback. The experimental settings of the trials other than the perturbation and probe trials (blue area in Figure 2B) were the same as those of the initial 100 trials.

In the *delay condition*, the participants performed a normal reaching task in which a 200-ms delay was introduced between the hand and cursor movements. After the initial 100 trials for habituation to the 200-ms delayed cursor, visual perturbation trials were randomly interleaved in the same manner as that during the *no-delay condition*. The perturbation and probe trials were the same as those observed during the *no-delay condition*. The experimental settings of the trials other than the perturbation and probe trials (red area in Figure 2B) were the same as those for the initial 100 trials of the *delay condition*.

All participants were tested in both the *no-delay* and *delay conditions*. Participants were randomly assigned to two groups. The first group (14 participants) performed the *no-delay condition* first, whereas the second group (13 participants) performed the *delay condition* first. They performed each condition within approximately 25 min without a break. Between the two conditions, participants took a rest for 10–20 min according to their fatigue.

It should be noted that, even in the *no-delay condition* (Figure 2D left), there was actually a 60 ms system delay between the handle movement and cursor position movement because of the data processing time of the computer (for more details, see Honda et al., 2012). Nevertheless, for clarity, we refer to this cursor as the *no-delay condition* in the sense that there is no additional delay other than the experimentally unavoidable delay, and the delay values of 100, 200, and 300 ms represented the additional delays (e.g., 100 ms indicated 160-ms delay).

### Data analysis

As a performance measure, we calculated the hand movement direction for each trial as the direction from the starting position to the actual hand position at the PV during the reaching movement. Baseline of the movement direction was calculated for each participant and for each condition by averaging the movement directions of 10 trials from 90th to 100th trial (the last 10 trials of the habituation trials).

We quantified the adaptation response to visual perturbation by measuring the aftereffect in the probe trial. The magnitude of aftereffect was calculated as the movement direction in the probe trial subtracted by the baseline value. The sign of the aftereffect was defined positive if the movement direction was opposite to the visual perturbation: the compensatory direction for the observed error. The data for the two perturbation directions was pooled. In cases when the reaching in the previous perturbation trial was “FAST,” “SLOW,” or deviated more than 10° from the target direction, we excluded the probe trial from data analysis. On an average, 21.2 ± 2.2 trials out of 24 (approximately 90%) in each *delay* or *no-delay condition* (6 trials × 4 amount of delay) satisfied the criteria.

In addition to the movement direction, the visual perturbation may also affect other movement parameters such as reaction time (RT) and PV in the next probe trial. The RT and PV were also calculated for each probe trial. The RT was calculated as the first time point at which the hand velocity exceeded 5% of the PV of that trial.

### Statistical analysis

Two-way repeated measures analysis of variance (ANOVA) was performed on the aftereffect, RT, and PV to examine the effects of cursor delay and condition. Ryan’s multiple comparison tests and one-way repeated measures ANOVA were used for *post hoc* analyses. The statistically significance threshold was set at *P* < 0.05.

## Results

**Figure 3:**
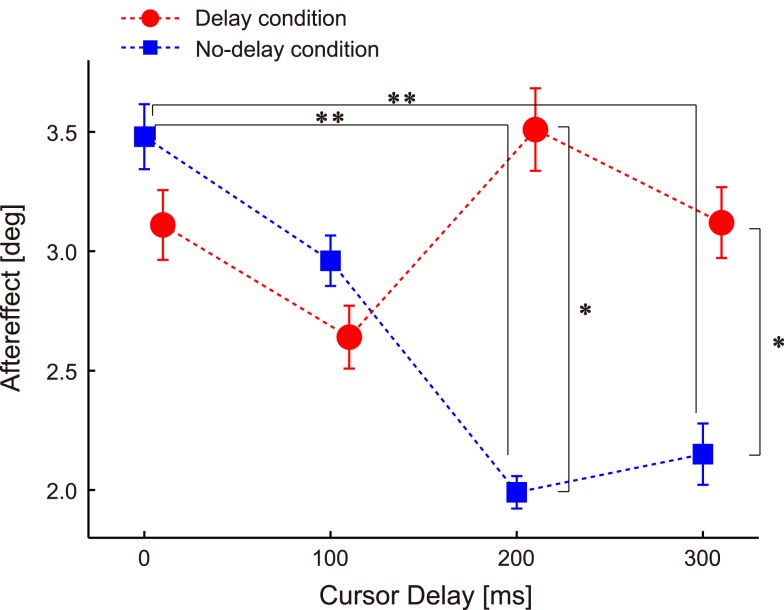
**Aftereffect of ±10° visuomotor rotation**. Data are sorted according to the cursor delay in their previous perturbation trials. The double asterisks illustrate the significant difference between cursor delays revealed by Ryan’s multiple comparison test conducted for the aftereffects in the *no-delay condition*. The single asterisks illustrate the significant main effect of condition revealed by one-way repeated measures ANOVA conducted for each cursor delay. Error bars indicate standard error.

Figure 3 shows the adaptation responses (i.e., the aftereffect in the probe trials) to the perturbations applied with the four types of delays (i.e., 0-, 100-, 200-, and 300-ms delay) under the two experimental conditions (blue, *no-delay condition*; red, *delayed conditions*). Two-way ANOVA with repeated measures revealed a significant interaction [condition × cursor delay, *P* = 0.006, *F*(3,78) = 4.483], indicating that the effect of delay on adaptation response depended on whether subjects were habituated to 0- or 200-ms delay in advance.

The results of the *no-delay condition* showed that the aftereffect was the largest when the perturbation was applied with a 0-ms delay and gradually decreased as the delay increased (blue squares in Figure 3). Statistical analysis confirmed this observation; one-way repeated measures ANOVA showed a significant effect of delay [*P* = 0.004, *F*(3,156) = 4.586], and the *post hoc* multiple comparison test showed significant differences between the 0- and 200-ms delays (*P* = 0.003) and between the 0- and 300-ms delays (*P* = 0.009; Figure 3).

How were these adaptation responses influenced when they were measured after habituation to the 200-ms delay? If the sensitivity of adaptation to a sensory prediction error is increased after habituation to the 200-ms delay and only this factor affects the aftereffect (i.e., H1), then the adaptation response should be recovered during a 200-ms delay and peak when visual rotation is applied with the habituated 200-ms delay (Figure 1B; red). On the other hand, if the sensory prediction error itself is increased after habituation and only this factor affects the aftereffect (i.e., H2), then the adaptation responses should be recovered in the 200-ms delay, and the feature that the adaptation responses gradually decays with increasing delay (i.e., they are largest for 0-ms delay) should remain.

Although the aftereffect for the 200-ms delay was larger in the *delay condition* than in the *no-delay condition* [*P* = 0.001, *F*(1,104) = 11.286] as both hypotheses predicted (Figure 3), the results were not completely compatible with these hypotheses. There was no significant difference in the aftereffect for the 0-ms delay between the conditions [*P* = 0.418, *F*(1,104) = 0.661; red circles in Figure 3], which was contrary to ideas that the aftereffect should peak at the 200-ms delay (i.e., H1) and that the aftereffect should be largest at the 0-ms delay (i.e., H2). The results of one-way repeated measure ANOVAs also contradicted both hypotheses; the results of the analyses showed no significant effect of delay after habituation to the *delay condition* [*P* = 0.322, *F*(3,156) = 1.174], indicating that there was no significant peak at the habituated 200-ms delay. Rather, it was likely that such a flat relationship between the aftereffect and level of delay could support the H3 (magenta in Figure 1E).

In our experiment, each participant performed the *delay* and *no-delay condition* successively. The effect of the condition order was unlikely to affect the results. To examine the effect, we analyzed the aftereffect of *no-delay condition* obtained from those who performed *no-delay condition* first (*N* = 14) and that of *delay condition* obtained from those who performed the *delay condition* first (*N* = 13). The pattern of the relationship between the aftereffect and level of delay was similar to the pattern obtained from the data combined together (Figure 3). The aftereffect for the 200-ms delay was significantly larger for the *delay condition* than for the *no-delay condition* [*P* = 0.000, *F*(1,100) = 14.427], but no significant effect of delay was observed for the *delay condition* [*P* = 0.0659, *F*(3,75) = 2.501].

**Figure 4:**
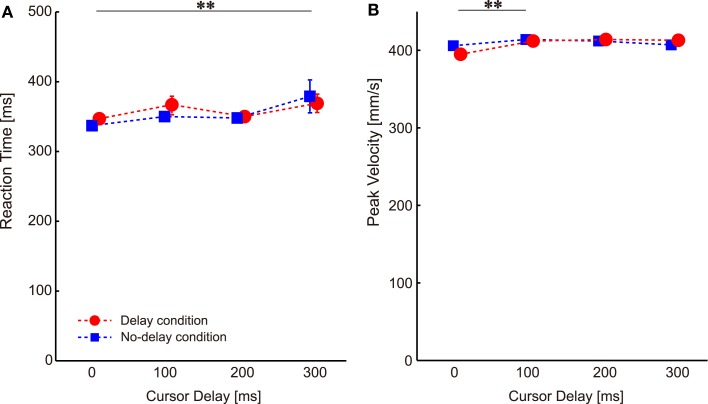
**Changes in the movement parameters throughout the experiments**. **(A)** Reaction time. **(B)** Peak velocity. Data are sorted according to the cursor delay in the previous perturbation trials. The double asterisks illustrate the significant difference between cursor delays revealed by Ryan’s multiple comparison tests. Error bars indicate standard error.

Visual perturbations might also affect other movement parameters such as RT and PV in subsequent probe trials. Two-way ANOVA with repeated measures on RT showed a significant main effect of cursor delay [*P* = 0.000, *F*(3,78) = 6.899], but no significant main effect of condition [*P* = 0.618, *F*(1,26) = 0.256] or interaction [condition × cursor delay, *P* = 0.462, *F*(3,78) = 0.866]. The multiple comparison across cursor delays showed a significant difference between the no-delay and the 300-ms delay (*P* = 0.042; the double asterisk in Figure 4A), indicating that visual perturbation applied with a 300-ms delay made the next movement take more time to initiate.

Two-way ANOVA with repeated measures on PV revealed a significant main effect of cursor delay [*P* = 0.007, *F*(3,78) = 4.360], but no significant main effect of condition [*P* = 0.744, *F*(1,26) = 0.109] or interaction [condition × cursor delay, *P* = 0.092, *F*(3,78) = 2.222]. The multiple comparison across cursor delay showed a significant difference between the no-delay and the 100-ms delay (*P* = 0.0498; the double asterisk in Figure 4B), indicating that visual perturbation applied with a short delay made the next movement faster.

These changes in RT and PV (gaps of less than 40 ms and 20 mm/s) were very small when compared with the large difference in RT (approximately 200 ms) and PV (approximately 50 mm/s) observed when visuomotor rotation was abruptly applied (Saijo and Gomi, 2010). We conclude that the RT and PV differences according to the cursor delay were not sufficient to explain the visuomotor aftereffect shown in Figure 3.

We also noted the reaching duration in the baseline trials under each condition, namely, 462 ± 24 ms for the *no-delay*, 476 ± 25 ms for the*delay condition*. For each of calculation, the reaching duration was calculated as the time until the hand velocity exceeded 5% of the PV of that trial. In 200- or 300-ms delayed cursor trials, a reaching duration shorter than 500 ms showed that the cursor was still moving halfway even when the reaching finished.

## Discussion

When a novel environment causes a discrepancy between the sensory prediction and actual sensory consequence, the sensory prediction error is used to correct the forward model (Shadmehr et al., 2010). Thus, in this motor learning scheme, the temporal relationship between the predicted and actual sensory consequences is important to accurately evaluate the sensory prediction error (Ikegami et al., 2012). As represented in tool manipulation, temporal relationships may vary, raising the suggestion that the brain can manage variable delays during motor learning. Contrary to this expectation, Tanaka et al. (2011) showed that even after participants habituated to a situation in which the endpoint of a reaching movement is visually provided with a certain amount of delay, the degradation of motor adaptation caused by feedback delay (see Kitazawa et al., 1995) was never restored. We presumed that the absence of a beneficial effect was due to the method of displaying the movement error. A recent study (Izawa and Shadmehr, 2011) showed that sensory prediction is not completely altered when only the endpoint is displayed (i.e., the forward model is not updated); rather, it is altered when the feedback of the entire movement path is provided. In accordance with this presumption, we recently succeeded in showing that after repeated exposure to the delayed cursor, the degraded motor adaptation of reaching movements is recovered (Honda et al., 2012), which indicates the ability of the motor learning system to adjust to the feedback delay variability.

Nevertheless, there are still several possibilities to explain this alleviation of visuomotor adaptation. As described in the Introduction, we raised 3 alternative hypotheses (H1, H2, and H3). This study was designed to examine which hypothesis could explain the data most appropriately. We systematically investigated how the adaptation response to visual rotation during reaching movements was dependent upon the amount of visual feedback delay after participants were habituated to either the 0-ms (*no-delay condition*) or the 200-ms delay (*delay condition*). In the *no-delay condition*, regardless of the hypotheses, the adaptation response (the aftereffect in the probe trial subsequent to the visual perturbation trial) should gradually decrease with increasing visual feedback delay as shown in previous studies (Kitazawa et al., 1995; Tanaka et al., 2011; Honda et al., 2012). This was confirmed by the data in the *no-delay condition* of the present study (Figure 3).

In the *delay condition*, the three hypotheses predict different patterns of the dependence of the adaptation response on the amount of visual feedback delay. Hypothesis H1 assumes that repeated exposure to a certain amount of feedback delay increases the sensitivity of the visuomotor adaptation to a certain amount of error. In this case, the adaptation response should be maximal when the feedback delay is the same as the habituation period (i.e., 200-ms delay in the red; Figure 1E). Conversely, hypothesis H2 predicts that the repeated exposure to a certain amount of feedback delay simply shifts the predicted hand position by forming an internal model of feedback delay much like a mechanism of Smith predictor (Miall et al., 1993). This shift contributes to the recovery of the degraded adaptation response to the visual feedback provided with 200-ms delay, because a larger sensory prediction error is experienced during the movement (Figure 1A vs. Figure 1D). However, since the sensory prediction error experienced during the movement should be larger as the feedback delay is smaller (Figure 1C vs. Figure 1D), we should observe a monotonic decrease in the adaptation response with the increase in delay (Figure 1E; blue). The hypothesis H3 that both mechanisms of H1 and H2 are involved predicts the pattern of the adaptation response that is the intermediate of that of H1 and H2 (Figure 1E; magenta).

The results of the present study demonstrate that the adaptation response to the visual rotation imposed with 200-ms delay increased in the *delay condition* as compared to that of the *no-delay condition* (Figure 3), which is consistent with the results of our previous study (Honda et al., 2012). On the other hand, there was no statistically significant difference in the adaptation response among the 0-, 100-, 200-, and 300-ms delays (Figure 3), suggesting that neither H1 nor H2 was likely to be supported by these results. Rather, hypothesis H3 was a more likely explanation of the results.

Thus, our interpretation of the results include the following: (a) habituation to delayed feedback shifts the predicted hand position toward the delayed cursor position, (b) the shift of the predicted hand position not only helps the brain quantify the sensory prediction error accurately, but also increases the sensitivity of the adaptation to a certain amount of error between the predicted and actual sensory consequences (i.e., sensory prediction error), and (c) these factors ultimately affect the degree to which the brain uses a sensory prediction error to correct a motor command in a subsequent trial through modifying the forward model. However, these changes do not necessarily indicate that the shift in predicted hand position is a phenomenon separate from the increase in the sensitivity of the adaptation. The adaptation response to a visual perturbation is not proportional to the amount of perturbation, but when the perturbation is large, the adaptation response tends to saturate or fall off (Wei and Kording, 2009). In other words, the proportional coefficient decreases with the amount of the perturbation (or sensory prediction error), implying that a large sensory prediction error is tightly coupled with a reduction in sensitivity to the adaptation to a certain amount of sensory prediction error.

We have assumed so far that only H1, H2, and H3 are alternative hypotheses. However, it should be noted that there remains another possibility to explain the present results. For example, the adaptation responses to no-delay cursor could remain large even after the habituation to 200-ms delay cursor, because no-delay environment is what we encounter most of the time. This would yield a bi-modal distribution of the adaptation curve over time delays, as is observed in the present study (Figure 3). In order to examine this point, future research should include much longer experimental studies.

A fundamental question is how the habituation to a *delay condition* contributes to the shift in the predicted hand position. It is still controversial that the brain constructs an internal model of the feedback delay itself (Miall et al., 1993). However, considering the ability of the brain to construct internal models of various dynamical systems (Shadmehr and Mussa-Ivaldi, 1994) and that delay in a system can be approximated by the continuous dynamical system (Dorf and Bishop, 2010), the brain might be able to form an internal model of the feedback delay. Further studies are required to clarify the validity of this hypothesis.

## Conflict of Interest Statement

The authors declare that the research was conducted in the absence of any commercial or financial relationships that could be construed as a potential conflict of interest.
